# Alexis Wound Retractor for Radical Cystectomy: A Safe and Effective Method for Retraction

**DOI:** 10.1155/2018/8727301

**Published:** 2018-12-09

**Authors:** Ajaydeep S. Sidhu, Eric Marten, Nikita Bodoukhin, George Wayne, Elizabeth Nagoda, Akshay Bhandari, Alan M. Nieder

**Affiliations:** ^1^Columbia University Division of Urology, Mount Sinai Medical Center, Miami Beach 33140, FL, USA; ^2^Herbert Wertheim College of Medicine, Florida International University, Miami 33199, FL, USA

## Abstract

Surgical site infection rates remain a common postoperative problem that continues to affect patients undergoing urologic surgery. Our study seeks to evaluate the difference in surgical site infection rates in patients undergoing open radical cystectomy when comparing the Bookwalter vs. the Alexis wound retractors. After institutional review board approval, we performed a retrospective chart review from February 2010 through August 2017 of patients undergoing open radical cystectomy with urinary diversion for bladder cancer. We then stratified the groups according to whether or not the surgery was performed with the Alexis or standard Bookwalter retractor. Baseline characteristics and operative outcomes were then compared between the two groups, with the main measure being incidence of surgical site infection as defined by the CDC. We evaluated those presenting with surgical site infections within or greater than 30 postoperatively. Of 237 patients who underwent radical cystectomy with either the Alexis or Bookwalter retractor, 168 patients were eligible to be included in our analysis. There was no statistical difference noted regarding surgical site infections (SSIs) between the two groups; however, the trend was in favor of the Alexis (3%) vs. the Bookwalter (11%) at less than 30 days surgery. The Alexis wound retractor likely poses an advantage in reducing the incidence in surgical site infections in patients undergoing radical cystectomy; however, multicenter studies with larger sample sizes are suggested for further elucidation.

## 1. Introduction

Self-retaining retractors fixed to the operating table have traditionally been used in major open operations. The Bookwalter retractor has been in use for several decades and is frequently used during major open operations, including open urologic surgery. More recently, combined wound-edge protectors and retractors are being used in operations at risk for surgical wound complications, including colorectal and gynecologic surgery [1, 2]. To our knowledge, the use of wound-edge protectors/retractors has not been studied in open urologic operations, though its feasibility was recently evaluated for robotic-assisted cystectomy [3].

The Alexis dual-ring wound protector/retractor (Applied Medical, Rancho Santa Margarita, CA) is a useful tool for open surgery including radical cystectomy with urinary diversion. The advantages of using this particular retractor include adequate and atraumatic wound retraction, protection from bowel contents and spillage, and potentially reduced incidence of surgical site infection (SSI).

We routinely use the Alexis retractor at our institution, where, in our experience, it provides adequate exposure during radical cystectomy. Though surgeon preference largely dictates retraction modality, it is important to consider whether such choices influence patient outcomes. Thus, we present our institution's initial perioperative outcomes while using the Alexis wound retractor for radical cystectomy and compare these cases to a similar cohort of cases using a traditional Bookwalter retractor.

## 2. Methods

After institutional review board approval, we performed a nonrandomized, retrospective chart review of 237 patients undergoing open radical cystectomy with a urinary diversion from February 2010 through August 2017. Of these, 168 patients were included in this study. Patients who underwent robotic cystectomy (15), simple cystectomy (11), and aborted cystectomies (11) were excluded from the analysis. In addition, 4 cases were excluded due to an intraoperative change to a different retractor. Finally, 28 cases were excluded due to insufficient data. The remaining 168 patients underwent radical cystectomy with a urinary diversion using either a Bookwalter or an Alexis wound retractor for the entirety of the operation. Different surgeons did indeed utilize the Alexis retractor in a nonrandomized fashion. Importantly, while the Bookwalter retractor was used predominantly in the first half of our radical cystectomy series, it was mostly utilized in our morbidly obese patients during the second half of the series when most of the surgeons had converted to the Alexis.

We evaluated the baseline characteristics of the two groups and compared their perioperative outcomes. Baseline characteristics included age, gender, history of diabetes, history of pelvic or abdominal radiation, smoking status, body mass index (BMI), and American Society of Anesthesiologists Physical Status Classification (ASA). All patients underwent preoperative medical clearance and, if indicated, cardiopulmonary clearance. Patients received a preoperative mechanical bowel preparation with polyethylene glycol or ERAS protocol. Our more contemporary patients received heparin 5000 units subcutaneously and oral alvimopan 12 mg preoperartively. All patients received intravenous antibiotic prophylaxis prior to incision with cefoxitin 2 g or clindamycin 900 mg as well as postoperative prophylaxis for up to 24 hours. Upon entering the peritoneum and reflecting the colon cephalad, the decision was made by the surgeon to use either the Alexis wound retractor or a traditional Bookwalter retractor. Fascial incisions were closed with a running loop 0-PDS suture (Ethicon), and primary skin closure was performed with staples.

Perioperative outcomes evaluated included estimated blood loss (EBL), operative time (OR time), type of urinary diversion performed (ileal conduit, orthotopic neobladder, or cutaneous ureterostomy), intraoperative complications, length of stay (LOS), and SSI stratified by time of presentation. SSI was defined using Centers for Disease Control (CDC) guidelines: “Any infection of the superficial or deep tissues or the organ/space affected by surgery, and which occurs within 30 days of surgery when no prosthesis is implanted” [4]. Differences between the two groups were assessed using standard statistical methods, including independent sample *t*-tests, Pearson chi-squared tests, and the Mann–Whitney *U*-test using RStudio version 1.0.153.

## 3. Results

We identified 168 patients who were included in our study. 95 operations were performed entirely with the Alexis retractor and 73 with the Bookwalter. Besides average BMI, baseline characteristics were similar between the two groups with no statistically significant differences (Table 1).

There was no statistically significant difference in the incidence of SSI between the Alexis and Bookwalter retractor groups (Table 2). There were no differences in surgical site infections at either more than 30 days or less than 30 days postoperatively. Overall, 9 (12%) patients in the Bookwalter group and 4 (4%) patients in the Alexis group presented with a SSI. A statistically significant difference was observed regarding operative time: the mean operative time for those patients in the Alexis vs Bookwalter cohort was 219 min vs. 242 min, respectively (*p* < 0.05). We also identified a median length of stay in favor of the Alexis vs Bookwalter cohort, 5 vs 6 days (*p* < 0.05). Also, ASA score was lower for the Alexis group.

## 4. Discussion

In our single-center, retrospective study, we found that the Alexis provides appropriate exposure during radical cystectomy, with a decreased operative time and a possible trend towards reduced wound infection. The Alexis wound retractor/protector was developed in 2000 and combines wound retractor with wound barrier protection. It is comprised of two plastic rings with a plastic sleeve connecting the rings. The inner ring is placed underneath the body wall, and the outer ring is placed on top of skin. The plastic sleeve is rolled over the outer ring to retract the body wall circumferentially outward, theoretically applying equal force throughout the wound.

The Alexis wound retractor is currently and frequently used at our institution for performing open cystectomies (as well as during extracorporeal diversions during robotic cystectomies) with outcomes comparable to those operations where a Bookwalter retractor is used. Combined retractors and wound-edge protectors, such as those incorporated in the design of the Alexis wound retractor, have been studied in other open abdominal operations, particularly in colorectal and obstetric/gynecologic procedures [1, 2]. The authors from these fields have reported mixed results with such technologies, but their studies appear to lend overall support to the anecdotally efficient and adequate anatomic exposure, improvement in SSI rate, and reduction in trauma to retracted tissues. SSIs are an important cause of morbidity following radical cystectomy with urinary diversion. This is especially concerning in obese and diabetic patients who often suffer from comorbid metabolic and immunologic impairment, as well as in patients with a history of radiation or smoking.

Clean-contaminated (Class II) operations as a category, which includes cystectomy with diversion, have an overall SSI rate of 3–11% [5]. Lavallée et al. reported the incidence of SSI after radical cystectomy to be 8% [6]. In our study, while there was a trend towards lower SSI between the Alexis and Bookwalter retractor groups (4% and 12% total, respectively), this difference was not statistically significant. The Alexis retractor may indeed provide the benefit of lower SSI rates; however, further studies are needed with more patients to confirm this finding. In addition, it appears that the Alexis retractor may provide the advantage of less operative time and be associated with a lower length of hospital stay. Several nonurological studies have produced similar findings in regards to postoperative wound infection rates when using the Alexis wound retractor. Mihalijevic et al. demonstrated a reduction in superficial SSI when using Alexis wound retractors in open abdominal surgery [7]. Further, Reid et al. demonstrated a reduced incidence of surgical site infection with the use of barrier retraction wound protection in colorectal surgery [8]. Within the colorectal surgery literature, Cheng et al. [2] found that the use of the Alexis retractor has an absolute risk reduction of SSI of 20% and that the use of 5 Alexis O-ring wound retractors is needed to prevent one probable SSI. Scolari Childress et al. [1] demonstrated significantly fewer episodes of uterine exteriorization with the use of the Alexis wound retractor, facilitating hysterectomy repair in situ, which the authors suggest may be due to improved surgical visualization using the retractor. Likely, such improved visualization contributes to the shorter operative times observed when using the Alexis retractor in our practice. Hinkson et al. [9] reported a significantly lower incidence of SSI in the Alexis group, which the authors postulate is due to the circumferential coverage the device offers, thus protecting the wound edges. Another study involving Huynh et al. [3] reviewed 15 cases where the Alexis retractor was used for the bowel diversion, and did not report any wound-related complications. Though their study had a limited sample size, the authors did draw similar conclusions regarding the safety and efficacy of using the Alexis retractor as opposed to traditional retraction methods.

Though our study does not demonstrate a statistically significant difference in wound infection rate between Alexis and Bookwalter retractors, our results do suggest that infection rates favor the use of the Alexis wound retractor. Given our smaller cohort, in combination with the relatively low rates of SSI, we were not able to detect a statistical difference in our study, as it was likely underpowered. Anecdotally, we have noted retractor setup and breakdown to be much easier than with fixed body-wall retractors. We have also found it to be faster and easier for our OR staff when counting instruments. As surgeons strive to contain operative costs, decreasing operative time is advantageous. In our study, the operative time of the Alexis cohort was 23 minutes shorter compared to that of the Bookwalter cohort. This time saved is likely related to multiple causes including increased surgeon experience, though we do believe that the Alexis setup and removal do indeed save time compared to a standard fixed retractor.

Our study does have significant limitations: most importantly that it is a single-center, non-randomized, and retrospective study. Multiple unknown biases could have thus affected which cases utilized which retractors. As mentioned, most contemporary patients in our study underwent radical cystectomy with the Alexis retractor. Therefore, our improved results in the Alexis cohort may indeed have been related to improved surgeons' experience. Finally, retraction in very obese patients is always difficult and thus standard retractors are typically used, at least in our experience.

## 5. Conclusion

The Alexis retractor is a safe and effective tool for retraction and wound protection during open radical cystectomy and may potentially reduce wound-related complications, improve operative time, and lead to lower length of stay among patients. The Alexis retractor likely provides the benefit of lower surgical site infections compared to traditional retraction methods; however, further, larger, prospective multicenter trials should be initiated to confirm our findings.

## Figures and Tables

**Table 1:** Patient demographics for those undergoing radical cystectomy with either the Bookwalter retractor or the Alexis retractor.

	Alexis		Bookwalter		*p* value	
Age (years), SD	73	8	71	9	0.23	^*∗*^
Gender					0.28	^*∗∗*^
Male	76	80%	63	86%		
Female	19	20%	10	14%		
BMI mean (kg/m^2^), SD	25.9	4.2	27.6	4.4	0.027	^*∗*^
BMI count 25–29.99	60	63%	51	70%	0.60	^*∗∗*^
BMI count >30	17	18%	19	26%	0.31	^*∗∗*^
History of diabetes	17	18%	18	25%	0.40	^*∗∗*^
Current smokers	14	15%	4	5%	0.12	^*∗∗*^
Prior						
Radiation/brachytherapy	6	6%	11	15%	0.12	^*∗∗*^

^*∗*^Independent sample *t*-test, ^*∗∗*^Pearson's chi-squared test, ^*∗∗∗*^Mann–Whitney *U*-test.

**Table 2:** Operative measures and outcomes for patients undergoing radical cystectomy with either the Alexis retractor or the Bookwalter retractor.

	Alexis		Bookwalter		*p* value	
ASA, SD	2.7	0.5	2.8	0.4	0.034	^*∗∗∗*^
EBL (mL), SD	386	241	375	157	0.77	^*∗*^
OR time (min), SD	219	42	246	60	0.0004	^*∗*^
Diversion						
Radical cystectomy/IC	92	97%	66	90%	0.82	^*∗∗*^
Radical cystectomy/neobladder	1	1%	5	7%	0.11	^*∗∗*^
Radical cystectomy/cutaneous ureterostomy	2	2%	2	3%	1.00	^*∗∗*^
Median LOS (days)	5		6		0.027	^*∗∗∗*^
SSI						
<30 days	3	3%	8	11%	0.092	^*∗∗*^
30–60 days	1	1%	1	1%	1.00	^*∗∗*^

## Data Availability

The data used to support the findings of this study are available from the corresponding author upon request.
